# Catalytic Pyrolysis of Coconut Fiber with MgCl_2_: Enhancing Thermal Degradation Kinetics and Product Selectivity

**DOI:** 10.1021/acsomega.5c09940

**Published:** 2026-02-02

**Authors:** Adjentina Benigna de Lima Spirandeli, Beatriz Silvério, Taisa Shimosakai de Lira, Thiago Padovani Xavier, Mario Sérgio da Luz, Kássia Graciele Santos

**Affiliations:** † 74348 Universidade Federal do Triângulo Mineiro, Uberaba, Minas Gerais 38064-200, Brazil; ‡ 680695 Universidade Federal do Espírito Santo, São Mateus, Espírito Santo 29932-540, Brazil

## Abstract

Coconut fiber (CCF), a lignocellulosic biomass, presents significant potential for renewable energy production through pyrolysis. This study investigates the catalytic pyrolysis of CCF in a fixed-bed reactor, focusing on the effects of temperature (623–723 K) and magnesium chloride (MgCl_2_) concentration (0–10%) on product yields and properties. Proximate and elemental analyses were used to characterize CCF’s composition, while thermogravimetric analysis (TGA) at heating rates of 20–50 K/min assessed thermal degradation kinetics using the Reparametrized Global Reaction (RGR) model. The kinetic analysis confirmed that MgCl_2_ reduced activation energy from 56.3 kJ·mol^–1^ to 29.3 kJ·mol^–1^, enhancing devolatilization efficiency. Pyrolysis experiments yielded bio-oil, biochar, and gas in the ranges of 47.4–52.4%, 29.7–37.2%, and 15.4–17.9%, respectively, depending on operating conditions. Higher temperatures increased bio-oil yield, peaking at 52.36% at 723 K without MgCl_2_, while 10% MgCl_2_ enhanced biochar production to 58.65% with fixed carbon contents up to 62.0% and higher heating values ranging from 22.4 to 25.1 MJ·kg^–1^. Gas chromatography–mass spectrometry (GC–MS) showed that MgCl_2_ promoted aldehyde formation (e.g., furfural) via hemicellulose dehydration, whereas higher temperatures favored phenolic compounds from lignin degradation. These findings highlight MgCl_2_’s role in tailoring pyrolysis pathways for optimized bio-oil and biochar production, offering insights into sustainable biomass conversion for energy and carbon sequestration applications.

## Graphical abstract

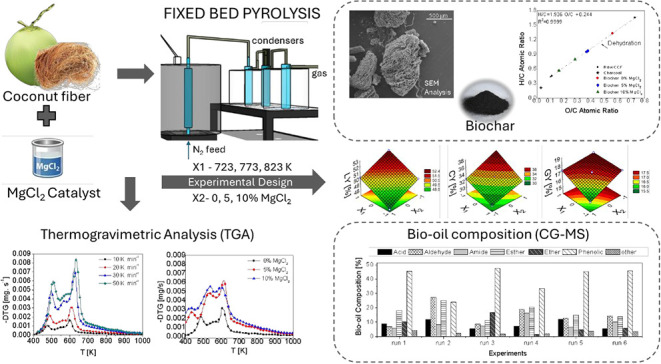

## Introduction

The increasing demand for sustainable energy solutions has intensified research into biomass conversion technologies, aiming to mitigate the environmental impacts of fossil fuels.^1,2^ Among these, pyrolysis has emerged as a promising thermochemical route for transforming lignocellulosic waste into valuable products such as bio-oil, biochar, and syngas.^3^ Coconut fiber (*Cocos nucifera* L.) is a lignocellulosic residue abundantly generated in tropical regions, where its slow natural degradation presents both environmental and solid-waste management challenges. Owing to its relatively high lignin content, this material is a promising feedstock for thermochemical conversion, particularly pyrolysis, enabling the production of biofuels and stable carbonaceous solids with potential for long-term carbon sequestration.^4^ From an economic standpoint, coconut coir fiber (CCF) is also a low-cost and readily accessible biomass. Brazil alone generates several million tonnes of coconut waste per year, the majority of which lacks commercial use and is typically discarded at minimal or no cost to industry or research institutions. As a result, collection expenses are largely limited to transportation and basic handling operations. This combination of abundant availability, continuous generation in both urban and agro-industrial settings, and negligible acquisition cost makes CCF an economically attractive feedstock for catalytic pyrolysis and decentralized biorefinery schemes.

Several studies have characterized the pyrolytic behavior of coconut shell under different operational conditions. Bandyopadhyay et al.^5^ performed pyrolysis in a tubular reactor and highlighted the feasibility of using coconut shell for liquid fuel production. Dhar et al.^6^ reported that pyrolysis of coconut fiber at higher temperatures (723–823 K) yields biochar with greater porosity and recalcitrance, favoring applications in soil amendment and carbon sequestration. In contrast, lower temperatures (623–723 K) produce biochar with higher volatile content and heating value, making it more suitable for use as solid fuel. Ahmad et al.^7^ examined the pyrolysis of coconut shell in a fixed-bed reactor, focusing on temperature and heating rate, as well as the impact of catalysts such as zeolite and dolomite. They found that optimal conditions for bio-oil yield (48.03%) were achieved at a pyrolysis temperature of 723 K, a heating rate of 50 K/min, and a particle size of 300 μm. Agrizzi et al.^8^ investigated the pyrolysis of green coconut shell and identified that optimal conditions at 773.15 K with a particle size of 1.3 mm resulted in a maximum bio-oil yield of 49.45%, rich in aldehydes, phenols, and esters, with temperature being the primary factor influencing their formation.

To enhance pyrolysis efficiency and product selectivity, the incorporation of catalysts has become a subject of growing interest. Specifically, alkaline earth metal salts such as magnesium chloride (MgCl_2_) have demonstrated the potential to modify pyrolysis pathways,^9^ lower activation energy barriers,^10^ and tailor product distributions.^11,12^ According to Khelfa et al.,^9^ MgCl_2_ has been shown to promote dehydration reactions, particularly influencing hemicellulose degradation and furfural formation. In the pyrolysis of malt waste^10^ and soybean hulls,^13^ MgCl_2_ increased aldehyde yields and altered the distribution of phenolics and acids, thereby affecting both the composition and quality of the resulting bio-oils.

Alkali and alkaline-earth metal chlorides such as NaCl, ZnCl_2_, and MgCl_2_ are known to influence biomass pyrolysis by promoting dehydration, shifting devolatilization temperatures, lowering activation energy, and modifying the distribution of oxygenated compounds.^9,10,13^ Among these salts, MgCl_2_ has shown a particularly distinctive catalytic behavior: as demonstrated by Santana et al.,^13^ it substantially reduces decomposition temperatures and enhances dehydration and deoxygenation pathways more effectively than NaCl, while causing less extensive cracking than strongly acidic salts like ZnCl_2_. These comparative trends suggest that MgCl_2_ provides a balanced catalytic effect, sufficient to accelerate devolatilization and oxygen removal without inducing excessive fragmentation, which supports its selection as the catalyst of interest in this work.

Besides, no previous study has evaluated the catalytic influence of MgCl_2_ on coconut coir fiber (CCF), a feedstock with unique composition and high availability, nor integrated kinetic modeling, fixed-bed pyrolysis, and GC–MS compositional analysis within a factorial experimental design. The present study addresses these gaps by systematically assessing how MgCl_2_ modulates both the thermal degradation behavior and the product selectivity of CCF pyrolysis. This is essential for optimizing the pyrolysis process to maximize bio-oil or biochar yield and enhance the economic viability of coconut fiber as a renewable resource.

The objective of this study was to evaluate the catalytic pyrolysis of coconut coir fiber (CCF) in a fixed-bed reactor, with emphasis on the effects of magnesium chloride (MgCl_2_) loading and pyrolysis temperature on the thermal degradation behavior, reaction kinetics, and the yield and composition of the resulting bio-oil and biochar. The research combines thermogravimetric analysis (TGA) and kinetic modeling to evaluate how the catalyst modifies thermal degradation behavior. Additionally, the study characterizes the chemical composition of bio-oil via GC-MS and assesses the physicochemical properties of biochar, providing insights into the potential applications of these products in energy, material, and environmental sectors.

## Material and Methods

### Biomass and Biochar Characterization

In this work, Magnesium chloride (MgCl_2_·6H_2_O) used for catalyst impregnation was an analytical-grade reagent (Synth, Brazil, ≥99% purity).

Coconut fiber (CCF) was obtained from the mesocarp of the coconut fruit after removing the epicarp, solid albumen, and endocarp. Coconut fiber was manually shredded, dried at 330 K for 48 h, and milled using a knife mill (Fortinox Star FT50, Fortinox, São Paulo, Brazil). The ground material was then sieved, and particles within the size range of 355 μm to 1 mm were selected to ensure uniformity in heat and mass transfer during pyrolysis experiments. The apparent density of the biomass was determined by liquid pycnometry using petroleum ether as the displacement fluid, while the bulk density was obtained by loose packing of the material into a graduated cylinder under gravity. The true density was measured by gas pycnometry using nitrogen (N_2_).

Proximate analysis of CCF biomass and biochar followed ASTM standards. Moisture content was determined on a wet basis (ASTM E871), extractives were quantified per ASTM D1105, and volatile matter (VM), fixed carbon (FC), and ash content were measured according to ASTM E872–82 and ASTM E1755–01, respectively. All analyses were conducted in triplicate, with mean values reported.

The elemental composition of CCF biomass was analyzed using an elemental analyzer (PerkinElmer CHN/O 2400, PerkinElmer, Waltham, MA) to quantify carbon (C), hydrogen (H), and nitrogen (N). Oxygen (O) content was calculated by difference, accounting for ash content. For biochar, the elemental composition (C, H, O) was estimated using correlations for torrefied biomass as described by Nhuchhen,^14^ following eqs 1–3. Nitrogen content was determined by difference.

1 C=−35.9972+0.7698·VM+1.3269·FC+0.3250·ASH

2 H=55.3678−0.4830·VM−0.5319·FC−0.5600·ASH

3 O=223.6805−1.7226·VM−2.2296·FC−2.2463·ASH

Higher heating values (HHV) were calculated using proximate analysis data according to the correlation of Parikh et al.,^15^ eq 4.

4 HHV=0.356·FC+0.1559·VM−0.0078·ASH

The main lignocellulosic compounds in the CCF biomass were quantified by the methodology described by Morais et al.^16^ Fourier-transform infrared spectroscopy (FTIR) was used to identify the functional groups in the CCF biomass and biochar, using a Bruker α Platinum-ATR (Bruker, Ettlingen, Germany) over the range of 4000–400 cm^–1^, with a resolution of 4 cm^–1^ and 32 scans.

The surface morphology of the biomass and biochar was characterized utilizing a scanning electron microscope (TESCAN VEGA3 LM, TESCAN, Brno, Czech Republic) equipped with an Energy Dispersive Spectroscopy (EDS) microanalysis detector (INCA X-ACT Standard, Oxford Instruments, Abingdon, U.K.).

### Thermogravimetric Analysis (TGA)

Thermogravimetric analysis (TGA) was conducted using a Shimadzu DTG-60 analyzer (Shimadzu Corporation, Kyoto, Japan) in an inert nitrogen atmosphere at a flow rate of 50 mL/min. The CCF samples were analyzed with heating rates of 20, 30, 40, and 50 K/min, covering a temperature range from 378 ± 3 to 1173.15 K. Two samples of CCF loaded with 5% and 10% (w/w) of MgCl_2_ were also analyzed, at a heating rate of 20 K/min. The TGA curves, including weight loss (TG) and the derivative of weight loss (DTG), were obtained to investigate thermal degradation kinetics.

### Kinetic and Thermodynamic study

The Reparametrized Global Reaction Model (RGR) was used to describe the biomass degradation, which consists of a reparametrized Arrhenius-type equation.^17^ Kinetic parameters (*E*_a_ and the order *n*) were estimated using the Differential Evolution Algorithm.^18,19^

The thermodynamic parameters were obtained from thermogravimetric data.^20^ The enthalpy change (Δ*H*), Gibbs free energy (Δ*G*), and entropy change (ΔS) were determined using eqs 5–7

5 $$\Delta H=E_{a}-\mathrm{RT}$$

6 $$\Delta G=E_{a}+RT_{p}\ln\left(\frac{K_{b}T_{p}}{h\cdot A}\right)$$

7 $$\Delta S=\frac{\Delta H-\Delta G}{T_{p}}$$

where *K*_b_ is the Boltzmann constant (1.381 × 10^–23^ J.K^–1^), *T*_p_ is the peak temperature from the DTG curve, and *h* is the Planck constant (6.626 × 10^–34^).

### Fixed-Bed Pyrolysis Experiments

The experimental setup is illustrated in Figure 1. The pyrolysis reactor consisted of a quartz tubular reactor (dimensions: 20 cm length and 3 cm diameter), housed within an electric furnace with a power capacity of 3000 W. The furnace was equipped with a digital PID temperature controller (ANOVA-N1200-USB) to maintain precise temperature regulation. Nitrogen gas (N_2_) was introduced into the reactor, with the flow rate controlled by a flowmeter. The condensation system comprised three cold traps arranged in series, cooled with ice to condense the volatile products. Noncondensable gases were washed with water before being safely vented into the atmosphere.^21,22^

**Figure 1:**
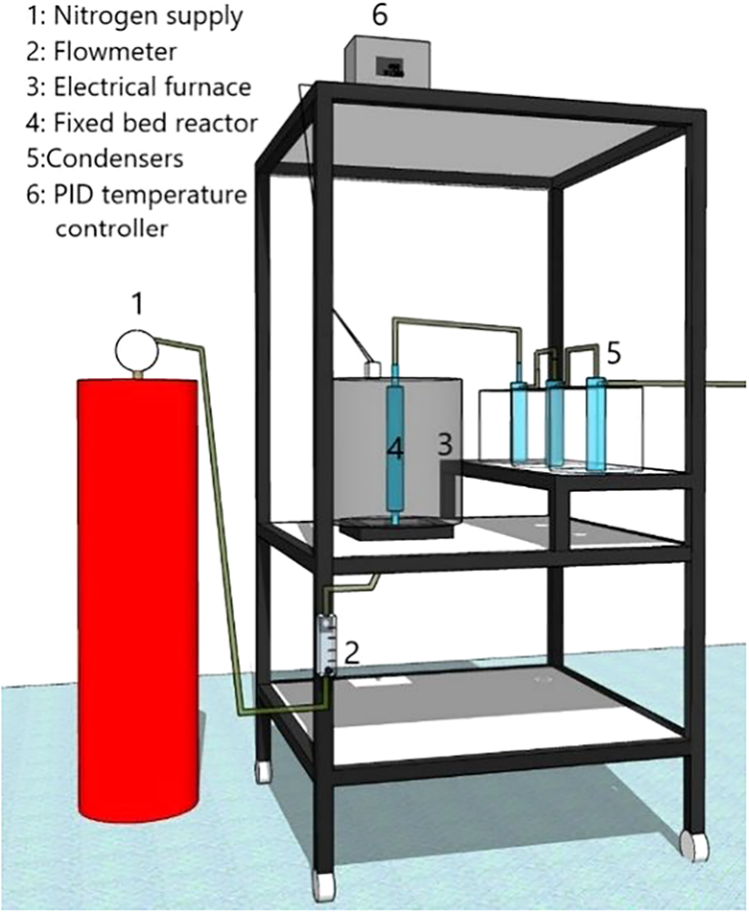
Experimental unit scheme of fixed-bed pyrolysis used in CCF thermal degradation.

The experiments in the fixed-bed reactor were conducted with a heating rate of 20 K/min and a nitrogen (N_2_) flow rate of approximately 100 mL/min. The system was maintained at the programmed pyrolysis temperature for each experimental condition (623, 673, or 723 K) for 30 min, according to the factorial design, followed by cooling to 333 K. The products yields were categorized into liquid (bio-oil and aqueous phase—LY), solid (biochar—CY), and gaseous (biogas—GY) fractions, calculated by eqs 8–10

8 $$\mathrm{LY}\left(\%\right)=\frac{W_{\mathrm{Liq}}}{w_{0}}\times 100\%$$

9 $$\mathrm{CY}\left(\%\right)=\frac{W_{\mathrm{char}}}{W_{0}}\times 100\%$$

10 GY(%)=100−LY−CY%

where *W*_Liq_ and *W*_Char_ are the masses of the condensed liquid and char, respectively, and *W*_0_ is the initial mass of the biomass.

A 2^2^ factorial design was employed to analyze the effects of pyrolysis temperature (*T* = 623 and 723 K) and MgCl_2_ mass fraction (*F*_A_ = 0 and 10%) on the product yields. Two central points (*T* = 673 K and *F*_A_ = 5% MgCl_2_) were included for replicates.

The coded factors for temperature (*X*_1_) and MgCl_2_ mass fraction (*X*_2_) are given by eqs 11 and 12, respectively

11 $$X_{1}=\frac{T-673\;K}{50\;K}$$

12 $$X_{2}=\frac{F_{A}-5\%}{5\%}$$

All responses were analyzed through multiple regression, calculating the curvature, linear, and interaction effects of independent factors (*X*_1_ and *X*_2_). The quality and accuracy of the model fitting were assessed using the coefficient of determination (*R* ^2^), the F-test (Fisher’s test). The statistical significance of the effects was evaluated using a significance level of α = 0.10, considering effects with p-value <0.10 as significant.

### Bio-Oil Characterization

To prepare samples for chemical composition analysis, the liquid phase collected from all traps was first homogenized and subsequently subjected to two sequential separation processes, according to Figure 2. The first step involved an acid fraction extraction, resulting in two distinct products: a black, viscous, water-insoluble fraction identified as crude bio-oil and an orange-yellow acid extract. A second extraction using dichloromethane as the solvent was performed to separate the coke. To eliminate residual coke and fine char particles, the organic solution was filtered through a 0.22 μm syringe. The final sample was analyzed via gas chromatography using a GC-MS-QP 2010 (Shimadzu Corporation, Kyoto, Japan) equipped with a flame ionization detector. The operational conditions for gas chromatography, including the column, oven temperature program, and mass spectrometry settings, were detailed by Agrizzi et al.^8^ and Moreira et al.^23^

**Figure 2:**
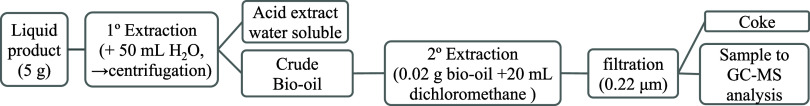
Scheme of extraction processes to obtain the sample for GC-MS analysis.

The chromatography data was processed using Automated Mass Spectral Deconvolution and Identification System (AMDIS) software (32-bit, version 2.65) to identify the compounds, with an emphasis on those with peak areas exceeding 1%. Compound identification was performed by comparing the spectra with the NIST08 library, considering a similarity index threshold of 80% or higher. The compounds identified in the bio-oil from each experiment were categorized based on their respective organic groups.^8^

## Results and Discussion

### Biomass Characterization

The characterization of coconut fiber (CCF) involved a detailed assessment of its physical and chemical properties, as summarized in Table 1. Elemental analysis revealed a carbon content (C) of 45.35 wt %, hydrogen (H) of 6.25 wt %, nitrogen (N) of 4.44 wt %, and oxygen (O) of 43.97 wt %, resulting in an H/C atomic ratio of 1.653 and an O/C atomic ratio of 0.727. These values indicate a relatively high oxygen content, which is typical for lignocellulosic biomass and suggests the potential for producing oxygen-rich bio-oils during pyrolysis. A high H/C atomic ratio signifies a greater degree of carbonization, along with increased aromaticity and condensation of the organic matrix within the biomass.^24^

**Table 1:** CCF Caracterization

elemental analysis [wt %]		chemical composition [wt %]	
C	45.35	extractives	9.34 ± 1.3
H	6.25	lignin	19.97 ± 1
N	4.44	cellulose	37.77 ± 0.15
O	43.97	hemicellulose	32.92 ± 0.51
H/C atomic ratio	1.653	cellulose/Lignin	1.891
O/C atomic ratio	0.727		

proximate analysis [wt %]		particle proprieties	
volatiles (VM)	85.42 ± 0.10	HHV [MJ/kg]	14.57
fixed carbon (FC)	10.83 ± 0.62	bulk density [kg/m^3^]	224.30 ± 0.9
ASH	3.75 ± 0.38	apparent density [kg/m^3^]	476.8 ± 2.5
		real density [kg/m^3^]	1246.8 ± 53.1

In the proximate analysis, the volatile matter (VM) content was 85.42 ± 0.10 wt %, while the fixed carbon (FC) accounted for 10.83 wt %, and the ash content was measured at 3.75 ± 0.38 wt %. The high volatile matter content suggests that CCF is highly reactive during thermal degradation, favoring the production of gases and bio-oil, while the relatively low ash content implies minimal mineral residue postpyrolysis.

The chemical composition showed that CCF contains 37.77 ± 0.15 wt % cellulose, 32.92 ± 0.51 wt % hemicellulose, and 19.97 ± 1 wt % lignin. The cellulose-to-lignin ratio was calculated to be 1.891, which reflects the high carbohydrate content, contributing to a significant potential for bio-oil production. Additionally, the extractives content was 9.34 ± 1.3 wt %, which may influence the yield and quality of biochar by introducing nonstructural components.

The higher heating value (HHV) of CCF was measured at 14.57 MJ/kg, which is typical for lignocellulosic materials and indicates moderate energy content for combustion applications.

### Kinetic and Thermodynamic Parameters from Thermogravimetric Analysis (TGA)

Figure 3a presents the devolatilization rate (DTG) curves as a function of temperature for heating rates of 10, 20, 30, and 50 K·min^–1^. The DTG profiles demonstrate that increasing the heating rate elevates the maximum conversion rate and produces more pronounced degradation peaks, reflecting the endothermic character of pyrolysis. At higher heating rates, the greater energy input accelerates biomass devolatilization.^25^ Moreover, all DTG curves shift slightly to higher temperatures, since rapid heating hinders uniform heat penetration within particles; as a result, shorter reaction intervals mandate higher temperatures to achieve thermal equilibrium and complete sample degradation.^26^

**Figure 3:**
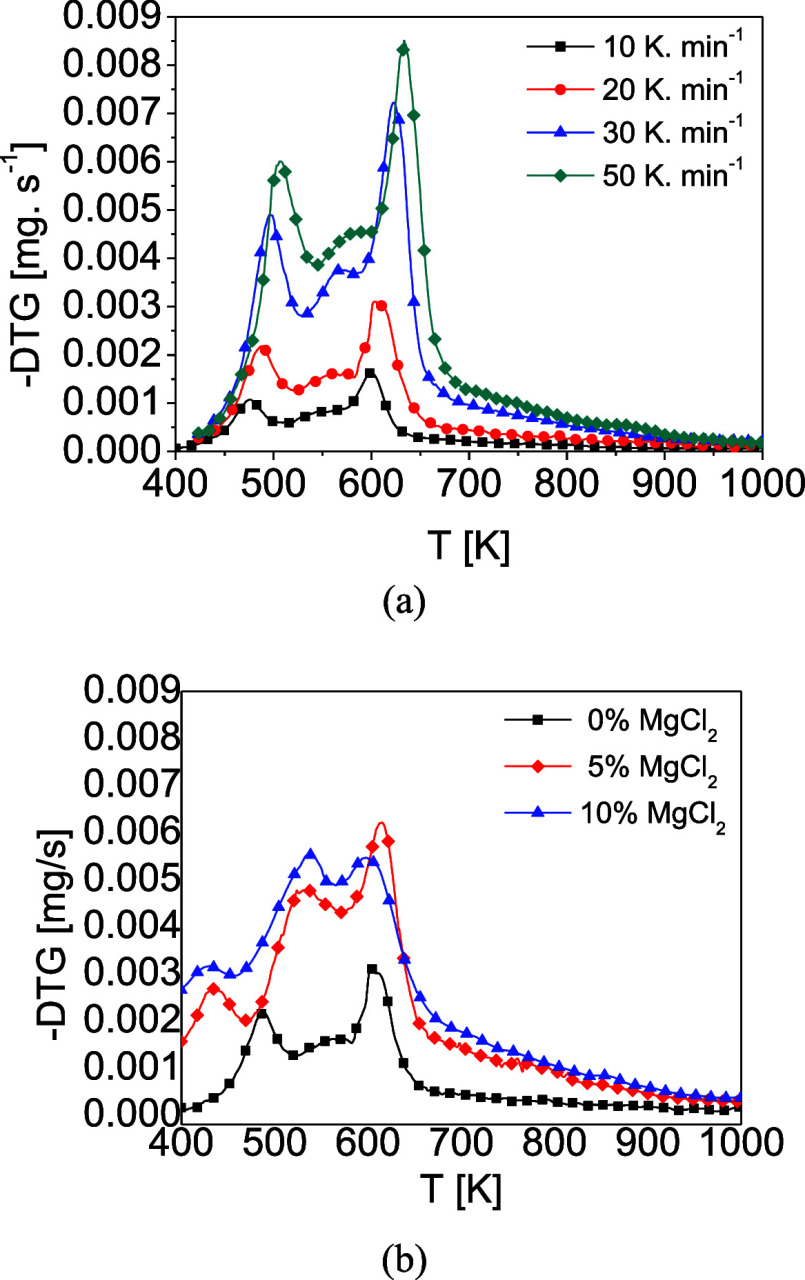
DTG curves: (a) Effect of heating rates and (b) Effect of MgCl_2_.

Between 410 and 520 K, the thermal degradation of extractives overlaps with hemicellulose decomposition,^27^ yielding an average conversion of ∼20%. A broader peak from 520 to 590 K corresponds to continued hemicellulose breakdown, contributing an additional ∼19% conversion. Taken together, extractives and hemicellulose account for ∼39% of the total mass loss, which agrees closely with the 36.3% fraction measured in the lignocellulosic analysis of the CCF sample.

The third distinct peak between 590 and 750 K is mainly associated with cellulose degradation, with an average conversion of ∼35%, which is of the same order of magnitude as the cellulose content determined by chemical analysis (37.77 wt %), indicating a consistent thermal signature. However, it should be noted that the conversion obtained from TGA reflects an apparent contribution of cellulose degradation within this temperature interval and does not represent a direct quantitative measurement of cellulose content, due to the overlap of degradation reactions and secondary char formation. In contrast, lignin undergoes a more gradual degradation over a wide temperature range (373–1173 K) and decomposes concurrently with other components.^28^

Figure 3b shows the effect of MgCl_2_ loading on the DTG curves of CCF at 20 K·min^–1^. Impregnation with MgCl_2_ shifts all degradation features to lower temperatures, indicating catalytic promotion of devolatilization.^29^ The most pronounced acceleration occurs in the extractives/hemicellulose region (458–561 K), whereas the cellulose peak (590–750 K) is less affected. This selective enhancement of low-temperature degradation corroborates observations by Carvalho et al.^30^ for sweet sorghum and Silva et al.^10^ for malt waste, both treated with MgCl_2_.

In nonisothermal reaction kinetics, single-reaction models are widely used to characterize the overall degradation rate of biomass. Table 2 summarizes the kinetic parameters of the RGR model derived from thermogravimetric data using the DE algorithm. The pre-exponential factors (*K*_0_) ranged from 1.85 × 10^3^ to 2.64 × 10^3^ s^–1^, while the activation energy values (*E*_a_) were between 58.5 and 55.5 kJ/mol. These *E*_a_ values align closely with those reported for coconut shell pyrolysis by Ashwini et al.^31^ (61.72 kJ/mol using ASTM E2070 method). Additionally, a decrease in Ea values was observed with increasing heating rates, consistent with findings by^17^ for sugar cane bagasse pyrolysis. The estimated reaction orders ranged from 1.35 to 1.85, reflecting the inherent complexity of the decomposition reactions.

**Table 2:** Kinetic and Thermodynamic Parameters of CCF Pyrolysis

kinetic parameter of RGR Model						
	noncatalytic-heating rate [K/min]				catalytic at 20 K/min	
parameters	10	20	30	50	5% MgCl_2_	10% MgCl_2_
*K*_0_ [s^–1^]	2.03 × 10^03^	1.85 × 10^03^	1.90 × 10^03^	2.64 × 10^03^	1.28 × 10^01^	2.59 × 10^00^
*E*_a_ [kJ mol^–1^]	58.5	56.3	55.7	55.5	36.4	29.3
N	4.45	3.91	3.22	2.85	2.64	2.45
FIT_TG_[%]	1.85	1.74	1.66	1.35	1.03	0.72
FIT_DTG_[%]	13.05	13.29	13.07	12.14	9.61	9.00
Thermodynamic Parameters						
*T*_p_ [K]	598.47	604.46	623.07	633.53	613.48	535.52
Δ*H* [kJ/mol]	5.35 × 10^01^	5.13 × 10^01^	5.06 × 10^01^	5.02 × 10^01^	3.13 × 10^01^	2.48 × 10^01^
Δ*G* [kJ/mol]	160.547	158.272	159.523	158.692	161.874	145.326
Δ*S* [kJ/mol]	–0.179	–0.177	–0.175	–0.171	–0.213	–0.225

As shown in Table 2, a notable decrease in activation energy (*E*_a_) was observed with increasing MgCl_2_ content: from 56.3 kJ·mol^–1^ (0% MgCl_2_) to 36.4 kJ·mol^–1^ (5% MgCl_2_) and 29.3 kJ·mol^–1^ (10% MgCl_2_). This reduction indicates that the catalyst lowers the energy barrier, allowing devolatilization to initiate at lower temperatures. The reduction in the pre-exponential factor (*K*_0_) in the presence of MgCl_2_ is consistent with a catalytic shift in the dominant decomposition pathways. As shown in Table 2, *K*_0_ decreases markedly from *K*_0_ = 1.85·10^3^ (20 K/min, 0% MgCl_2_) to *K*_0_ = 2.59 (20 K/min, 10% MgCl_2_), accompanying the drop in activation energy. In the Arrhenius framework, lower K_0_ values indicate that fewer high-energy molecular rearrangements are required to reach the activated complex, reflecting the ability of Mg^2+^ to promote dehydration and stabilize intermediate transition states. Concurrently, the fitted reaction order (n) decreases with higher MgCl_2_ content, suggesting that the degradation pathways become less complex, consistent with the findings reported by Silva et al.^10^ for MgCl_2_-catalyzed malt waste pyrolysis.

The reduction in the pre-exponential factor (*K*_0_) in the presence of MgCl_2_ also reflects a catalytic shift in the decomposition pathways. In the Arrhenius model, a lower *K*_0_ indicates that fewer high-energy molecular rearrangements are required for bond cleavage, consistent with Mg^2+^ facilitating dehydration and stabilizing intermediate transition states. This coordinated decrease in both *E*_a_ and *K*_0_ is characteristic of catalytic pyrolysis and signifies a modified reaction landscape rather than an experimental artifact.

The thermodynamic parameters at 20 K·min^–1^ (Table 2) further confirm the catalytic effect, which significantly reduced both *E*_a_ and enthalpy change (Δ*H*), with Δ*H* decreasing from 51.3 kJ·mol^–1^ for raw biomass to 24.8 kJ·mol^–1^ with the addition of 10% MgCl_2_. Once Δ*H* and *E*_a_ differ by less than 6 kJ·mol^–1^ in every case, the energy required for product formation is minimal, enhancing the reaction’s feasibility. The Gibbs free energy (Δ*G*) values 158.27 kJ·mol^–1^ (0% MgCl_2_), 161.87 kJ·mol^–1^ (5%), and 145.33 kJ·mol^–1^ (10%) remain positive, confirming that thermal degradation is nonspontaneous and heat-driven. These Δ*G* and Δ*H* values agree with those reported for raw coconut shell^32^ (Δ*H* = 65.2 kJ·mol^–1^, Δ*G* = 193.1 kJ·mol^–1^) and other biomasses.^10,23^ Finally, the negative entropy change (Δ*S*) values from −0.177 kJ·mol^–1^ (0% MgCl_2_) to −0.225 kJ·mol^–1^ (10% MgCl_2_) indicate a decrease in system disorder, implying that MgCl_2_ promotes a more ordered, efficient reaction network.^10^

#### Fixed-Bed Pyrolysis

Table 3 presents the fixed-bed reactor conditions and resulting product yields. A statistical analysis evaluated the effects of pyrolysis temperature and MgCl_2_ mass fraction on liquid, biogas, and biochar yields. Diagnostic tests confirmed that residuals were normally distributed, independent, zero-mean, and homoscedastic. Experimental variability was assessed using the two replicated central-point runs of the factorial design. The differences between duplicates were minimal (0.05% for bio-oil, 0.04% for biochar, and 0.01% for gas yield), corresponding to an average relative deviation of 0.09%. These results confirm the high reproducibility of the fixed-bed pyrolysis system and justify the use of single runs for the remaining factorial points.

**Table 3:** Experimental Factorial Design: Influence of Temperature (*T*) and MgCl_2_ Concentration (*F*_a_) on Product Yields and Biochar Characteristics

	factors		products yield			biochar characteristics							
run	*T* [K]	*F*_A_ [%]	LY [%]	CY [%]	GY [%]	ASH (%)	VC (%)	FC (%)	%C	%H	%O	%N	HHV [MJ/kg]
1	623	0	49.91	32.49	17.60	9.36	70.08	20.56	48.27	5.34	36.09	10.29	18.17
2	623	10	47.44	37.17	15.39	12.56	43.56	43.89	59.85	3.95	22.59	13.60	22.32
3	723	0	52.36	29.73	17.91	13.65	27.70	58.65	67.58	3.15	14.54	14.73	25.09
4	723	10	50.15	32.56	17.29	18.11	26.72	55.18	63.67	2.98	13.96	19.39	23.67
5	673	5	49.46	33.36	17.18	14.66	50.99	34.35	53.60	4.26	26.33	15.82	20.06
6	673	5	49.41	33.4	17.19	12.98	52.77	34.25	54.29	4.39	27.26	14.06	20.32

Table 4 presents the ANOVA results, while Figure 4 shows the corresponding response surfaces. Although the response surfaces were generated using a first-order (linear) model, the 2^2^ factorial design with center points enables only the detection of curvature, not its explicit modeling. Consequently, when statistically significant curvature was observed, the actual response behavior is likely nonlinear. To capture such nonlinear effects with greater accuracy, the experimental design would need to be expanded to include additional factor levels, such as a central composite design (CCD), allowing the explicit modeling of curvature effects.

**Figure 4:**
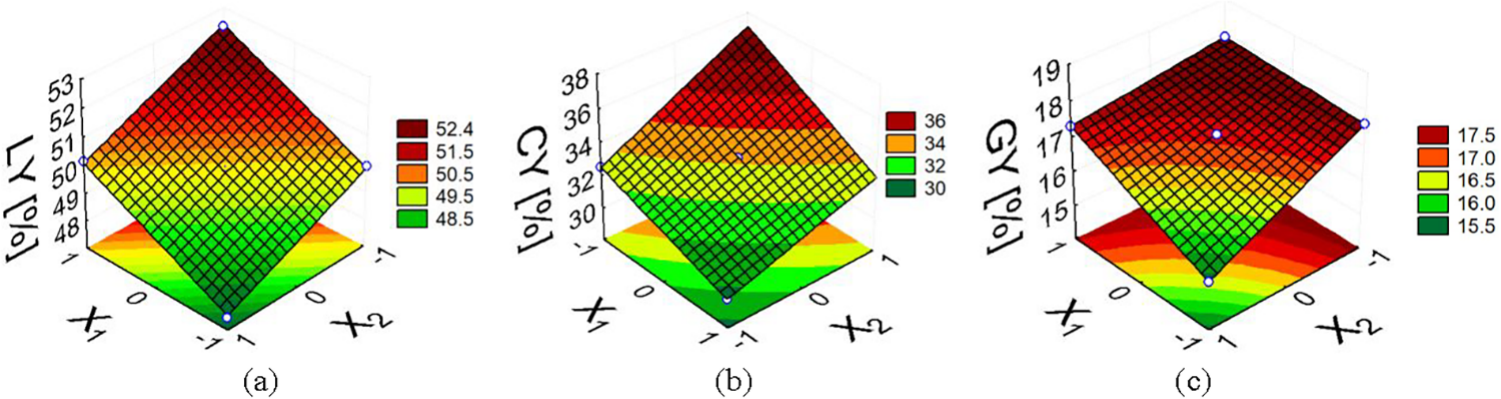
Surface responses of product yields as a function of temperature (*X*_1_) and MgCl_2_ concentration (*X*_2_): (a) liquid; (b) biochar, and (c) biogas.

**Table 4:** Effect of Pyrolysis Temperature (*X*_1_) and MgCl_2_ Concentration (*X*_2_) on Liquid, Biochar, and Biogas Yields

	liquid product yield			biochar yield				biogas yield		
factor	effect		*p*-value	effect			*p*-value	effect		*p*-value
mean	49.965	±0.018	<0.001	32.988	±0.014	<0.001		17.048	±0.035	<0.001
curvature	–1.060	±0.061	0.037	0.785	±0.049	0.040		0.275	±0.012	0.028
*X*_1_	2.580	±0.035	0.009	–3.685	±0.028	0.005		1.105	±0.007	0.004
*X*_2_	–2.340	±0.035	0.010	3.755	±0.028	0.005		–1.415	±0.007	0.003
*X*_1_ *X*_2_	0.130	±0.035	^a^0.169	–0.925	±0.028	0.020		0.795	±0.007	0.006
*R* ^2^	0.9998			0.9999				0.9999		

a Nonsignificant parameter.

For liquid yield (mean = 49.965%), both temperature (*X*_1_) and MgCl_2_ concentration (*X*_2_) were statistically significant (*p*-value <0.10). Increasing temperature enhanced liquid production, while higher MgCl_2_ loadings reduced it; thus, high temperature combined with low salt content favors liquid formation. Within the temperature range investigated in this study, the highest liquid yield (LY) was achieved at 723 K for noncatalytic pyrolysis, attributed to the increased energy available for breaking strong organic bonds. Conversely, the lowest LY was observed at 623 K with 10% MgCl_2_. These trends are evident in Figure 4a, where liquid yield rises with temperature and declines as MgCl_2_ concentration increases.

Many works reported a nonlinear influence of alkaline earth metal catalysts on bio-oil production during biomass pyrolysis,^10,12,33^ which justifies the significant curvature effect found in this work. According to Liu et al.,^11^ the presence of MgCl_2_ significantly influences the reaction mechanisms of cellulose. While the pyrolysis of pure cellulose primarily proceeds through depolymerization, releasing sugars, the addition of magnesium chloride shifts the process toward cross-linking reactions due to the weakening of hydrogen bonds.

The biochar yields were affected by both temperature and MgCl_2_ concentration (*p*-value <0.10), with an average yield of 32.988%. From Table 4, the negative sign of the main temperature effect, together with its greater magnitude relative to the interaction term, indicates that lower pyrolysis temperatures favored biochar formation. Meanwhile, the positive effect of MgCl_2_ concentration suggests that the presence of MgCl_2_ enhanced biochar formation, as illustrated by the response surface in Figure 4b, due to its catalytic role in promoting dehydration and carbonization reactions.^9^

Both temperature and MgCl_2_ concentration significantly (*p*-value <0.10) affected biogas yield (mean = 17.048%). Although higher temperatures generally favored biogas formation, the ANOVA revealed a significant interaction between temperature and MgCl_2_ loading, indicating that the effect of temperature depends on the catalyst level. As shown in Figure 4c, the lowest biogas yield was obtained at the lowest temperature combined with the highest MgCl_2_ concentration, evidencing the inhibitory role of the catalyst under mild thermal conditions. Nan et al.^34^ and Liu et al.^35^ reported that MgCl_2_ impregnation promotes the formation of a thin MgO–MgO_3_(CO_3_)_2_ layer on the biochar surface, which acts as a physical barrier limiting devolatilization and retaining carbon, thereby suppressing gas release. In addition, MgCl_2_ alters reaction pathways and product selectivity by promoting cellulose dehydration while leaving depolymerization and vaporization largely unchanged.^36^ According to Khelfa et al.,^9^ Since cellulose degradation is the primary source of light gases (CO and CO_2_), and MgCl_2_ also suppresses xylan degradation and CO_2_ formation at lower temperatures,^9^ the observed interaction reflects a combined thermal–catalytic control over gas evolution rather than a simple main-effect behavior.

#### Bio-Oil Characterization

Additional insights into the catalytic effects can be obtained by comparing the evolution profiles of the volatile products generated during thermal decomposition. Table 5 provides a list of components derived from the GC/MS profiles of the bio-oils across all experimental runs.

**Table 5:**
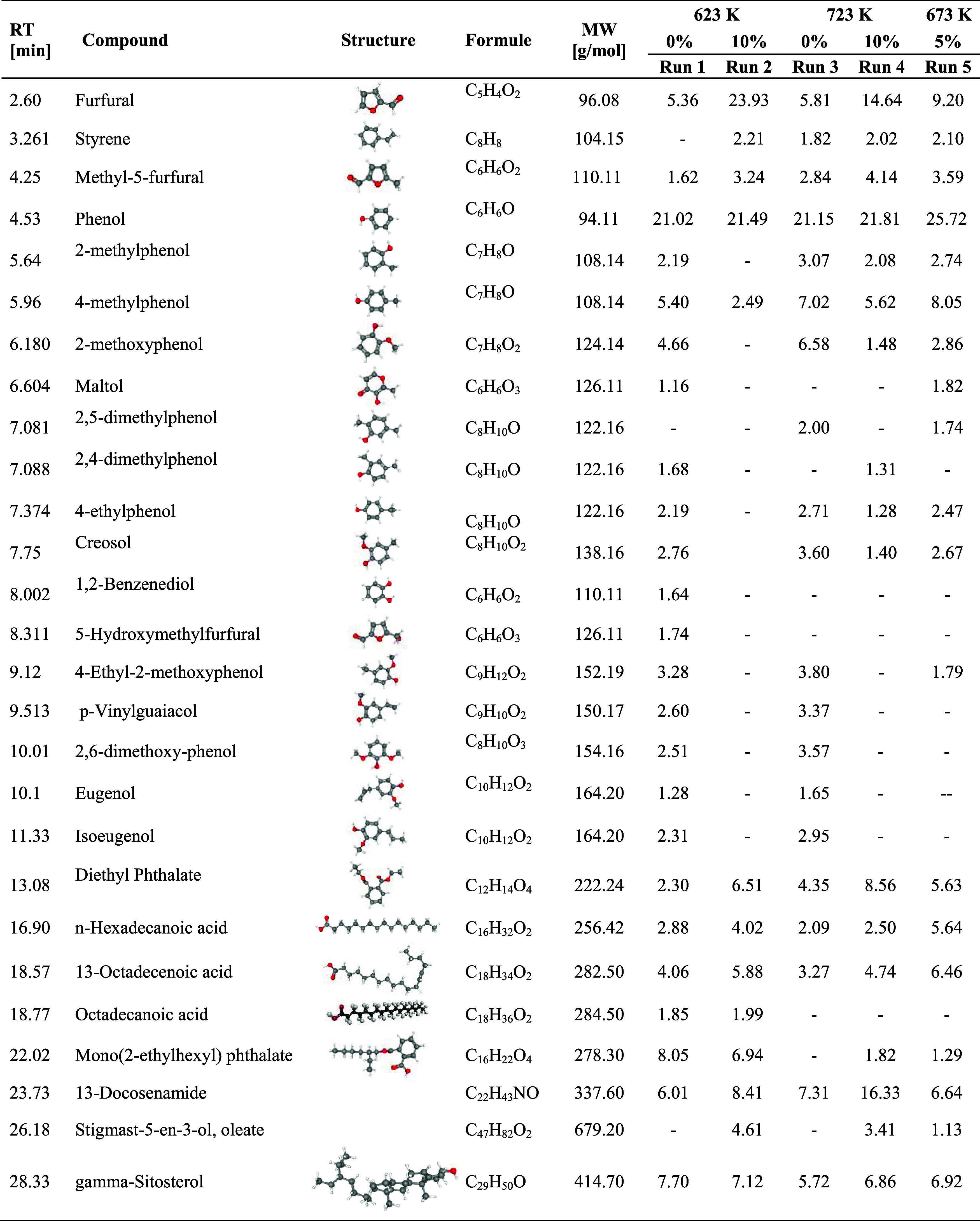
Compounds Identified in the Bio-Oil from CCF Pyrolysis

Figure 5 shows the major compounds identified in the CCF bio-oil for each run. Phenol was identified as the major compound in all bio-oil samples, except for Run 2 (723 K, 10% MgCl_2_). Its consistent presence across different experimental conditions suggests that the decomposition of lignin, a major component of CCF, is a primary source of phenolic compounds.^37^ The high phenol content aligns with the lignocellulosic composition of CCF, which contains 19.97 wt % lignin. Phenolic compounds are valuable due to their applications in the production of resins, adhesives, and other industrial chemicals.^38^ Almeida et al.^4^ and Agrizzi et al.^8^ also reported the phenolic compounds as the main component of bio-oil from coconut shell.

**Figure 5:**
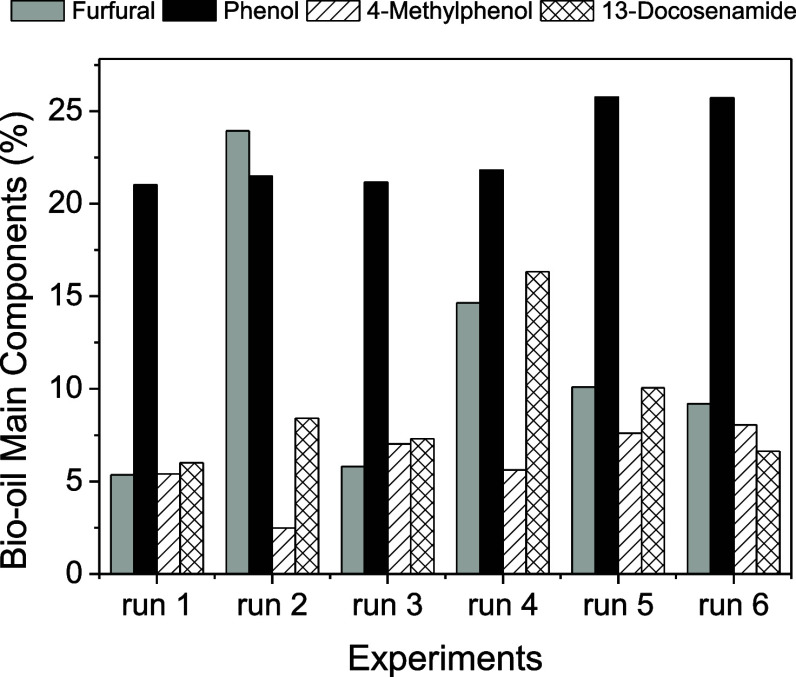
Major compounds identified in CCF bio-oils: Run 1­(*T* = 623 K, *F*_A_ = 0%); Run 2 (*T* = 623 K, *F*_A_ = 10%); Run 3 (*T* = 723 K, *F*_A_ = 0%); Run 4 (*T* = 723 K, *F*_A_ = 10%); Runs 5 and 6 (*T* = 673 K, *F*_A_ = 5%).

Furfural, an oxygenated compound derived from hemicellulose degradation,^39^ exhibited a marked increase during catalytic pyrolysis with 10% MgCl_2_, especially at 623 K. This suggests that MgCl_2_ promotes the dehydration of pentose sugars, enhancing furfural formation. Similar catalytic effects at low temperatures were reported by Liu et al.^40^ for cotton stalk pyrolysis. These findings align with previous studies showing that alkaline earth metal chlorides facilitate hemicellulose depolymerization under mild thermal conditions.^9,10^

The highest concentration of 13-Docosenamide, a long-chain amide, was observed at 723 K with 10% MgCl_2_ (Run 2). This compound is likely derived from the degradation of fatty acids or proteins present in the biomass. The formation of 13-Docosenamide at higher temperatures suggests that MgCl_2_ catalyzes the breakdown of complex organic structures, leading to the release of nitrogen-containing compounds. This finding is significant because nitrogen-containing compounds can influence the stability and quality of bio-oil. The presence of MgCl_2_ also influenced the formation of other oxygenated and aromatic compounds, such as methyl-5-furfural, 2-methoxyphenol, and eugenol. These compounds are typically derived from the decomposition of cellulose and lignin.

The identified compounds by the GC-MS technique were categorized according to their chemical classes for comparative analysis, as shown in Figure 6. The formation of acids and amides in the bio-oil derived from CCF pyrolysis was observed across all experimental conditions, with acid content ranging from 5.36% to 12.10% and amide content varying between 6.01% and 16.33%. No significant trend was observed in the formation of these compounds concerning either pyrolysis temperature or MgCl_2_ catalyst concentration.

**Figure 6:**
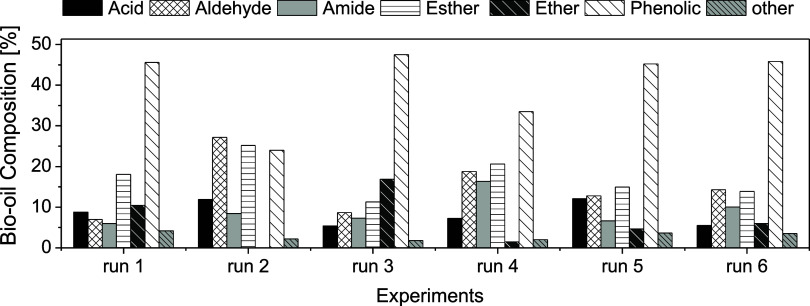
Analysis of product composition in CCF Bio-oil. The bar chart classifies products into acids, aldehydes, amides, esters, ethers, phenols, and other compounds, comparing their distributions across experimental conditions: Run 1 (*T* = 623 K, FA = 0%), Run 2 (*T* = 623 K, FA = 10%), Run 3 (*T* = 723 K, FA = 0%), Run 4 (*T* = 723 K, FA = 10%), and Runs 5 and 6 (*T* = 673 K, FA = 5%).

The response surface methodology (RSM) was applied to investigate the effects of temperature (*X*_1_) and MgCl_2_ concentration (*X*_2_) on the chemical composition of the bio-oil. Table 6 and Figure 7 present the influence of these factors on the yields of aldehydes, esters, ethers, and phenolics in the bio-oil.

**Figure 7:**
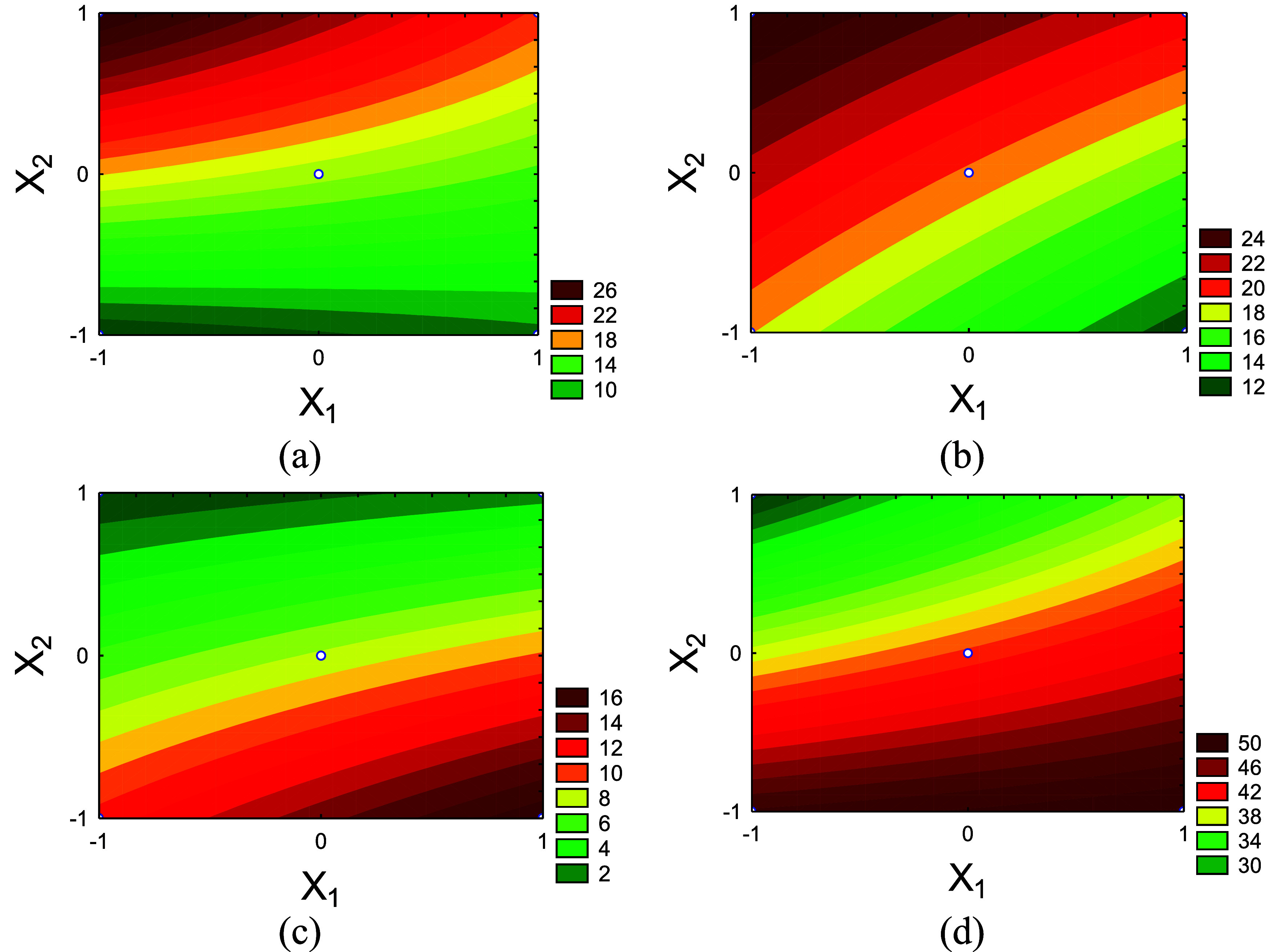
Contour plots showing the influence of temperature (*X*_1_) and MgCl_2_ concentration (*X*_2_) on the production of key compound groups in CCF bio-oil: (a) aldehydes, (b) esters, (c) ethers, and (d) phenols.

**Table 6:** Effect of Factor *X*_1_ (Temperature) and *X*_2_ (MgCl_2_ Concentration) on the Bio-Oil Composition

	aldehyde		esthers		eters		phenolics	
factors	effects	*p*-value	effects	*p*-value	effects	*p*-value	effects	*p*-value
mean	15.400	0.022	18.793	0.013	7.195	0.042	37.643	0.004
curvature	–3.720	^a^0.290	–8.745	0.097	–3.760	^a^0.260	15.735	0.030
*X*_1_	–3.360	^a^0.193	–5.645	0.087	3.940	^a^0.149	5.735	0.047
*X*_2_	15.160	0.044	8.245	0.060	–12.910	0.046	–17.805	0.015
*X*_1_ *X*_2_	–5.030	^a^0.131	1.115	^a^0.388	–2.460	^a^0.232	3.785	0.071
*R* ^2^	0.9959		0.9952		0.9954		0.9996	

a Nonsignificant parameter.

The formation of aldehydes in the bio-oil was significantly influenced only by MgCl_2_ concentration (*X*_2_) (*p*-value <0.10), while temperature (*X*_1_) did not present statistically significant main or interaction effects within the investigated range. The average aldehyde content was 15.40%, and the positive effect of *X*_2_ indicates that increasing MgCl_2_ loading promotes aldehyde formation. This behavior is consistent with the catalytic role of MgCl_2_, reported by Wan et al.,^12^ in promoting the dehydration of hemicellulose-derived sugars, particularly leading to the formation of furfural. The highest aldehyde yield was observed at 623 K and 10% MgCl_2_ loading (run 2).

The ester content in the bio-oil was between 11.29 to 25.18%. The temperature had a negative influence, suggesting that higher temperatures may lead to the decomposition of ester compounds. Conversely, MgCl_2_ concentration exhibited a positive effect, indicating that the catalyst promotes ester formation, likely through the stabilization of intermediate compounds derived from lipid degradation. The curvature effect was significant, hinting at a potential nonlinear relationship at extreme factor levels.

Ether formation was significantly influenced only by MgCl_2_ concentration (*X*_2_), which exhibited a strong negative effect (*p*-value <0.10). Although temperature showed a slight positive trend, its effect was not statistically significant within the studied range (*p* > 0.10). This suggests that the catalyst suppresses ether production, likely by redirecting reaction pathways toward the formation of alternative compounds such as aldehydes and phenolics. Consequently, the highest yield was observed at the highest temperature in the absence of the catalyst.

Phenolics constituted the predominant class of compounds in the bio-oil, with yields ranging from 23.98% to 47.52%. The analysis revealed that phenolics increased with temperature (*X*_1_), with this positive effect being dependent on MgCl_2_ concentration (*X*_2_), as evidenced by the significant and positive main and interaction effects. This can be attributed to enhanced lignin degradation at elevated temperatures. In contrast, MgCl_2_ concentration (*X*_2_) demonstrated a strong negative influence, likely due to the catalyst redirecting reaction pathways toward the formation of other compounds, such as aldehydes or gases. A significant curvature effect was observed, indicating that some nonlinear effects of factors could be significant.

So, while higher temperatures generally favored the formation of phenolics and ethers, MgCl_2_ concentration played a critical role in enhancing aldehyde and ester yields while suppressing phenolic and ether content. These findings highlight the complex interplay between process variables and reaction pathways during catalytic pyrolysis. These results were consistent with the findings reported by Santana et al.,^13^ who investigated the catalytic pyrolysis of soybean hulls using MgCl_2_, which reported an increase in aldehyde yields (e.g., furfural) while reducing the formation of acids and esters due to MgCl_2_ addition.

### Biochar Characterization

Table 3 provides the proximate analysis results for CCF biochar, which are used to estimate its elemental composition and higher heating value (HHV). Table 7 presents the statistical analysis of the quality responses of biochar.

**Table 7:** Effect of Factor *X*_1_ (Temperature) and *X*_2_ (MgCl_2_ Concentration) on CCF Biochar Quality Responses (Elemental Analysis, Proximate Analysis, and HHV)

biochar proximate analysis								
	ASH (*R* ^2^ = 0.965)		VC (*R* ^2^ = 0.999)		FC (*R* ^2^ = 0.999)		HHV (*R* ^2^ = 0.999)	
factors	effects	*p*-value	effects	*p*-value	effects	*p*-value	effects	*p*-value
mean	13.553	0.001	42.015	0.010	44.570	0.001	22.3125	0.003
curvature	0.800	^a^0.764	19.730	0.070	–20.540	0.004	–4.2450	0.048
*X*_1_	4.920	0.032	–29.610	0.027	24.690	0.002	4.1350	0.028
*X*_2_	3.830	0.051	–13.750	0.058	9.9300	0.005	1.3650	0.085
*X*_1_ *X*_2_	0.630	^a^0.557	12.770	0.062	–13.400	0.004	–2.7850	0.042

biochar elemental analysis								
	%C (*R* ^2^ = 0.999)		%H (*R* ^2^ = 0.998)		%O (*R* ^2^ = 0.999)		%N (*R* ^2^ = 0.956)	
factors	effects	*p*-value	effects	*p*-value	effects	*p*-value	effects	*p*-value
mean	59.843	0.003	3.855	0.008	21.795	0.010	14.658	0.001
curvature	–11.795	0.046	0.940	0.100	10.000	0.072	0.875	^a^0.755
*X*_1_	11.565	0.027	–1.580	0.037	–15.090	0.028	5.110	0.032
*X*_2_	3.835	0.081	–0.780	0.075	–7.040	0.059	3.985	0.052
*X*_1_ *X*_2_	–7.745	0.040	0.610	0.095	6.460	0.065	0.675	^a^0.551

a Nonsignificant parameter.

The proximate analysis revealed a high ash content (9.36 to 18.11%), largely attributed to the presence of the catalyst. An increase in both factors, temperature and MgCl_2_ concentration, contributes to an increase in the biochar ash content. Pyrolysis resulted in a significant increase in fixed carbon (FC) in the biochar (20.56–58.65%) compared to the raw CCF biomass (10.83%). According to Table 7, a more pronounced effect of temperature is observed in noncatalytic pyrolysis over the fixed carbon of biochar, due to the interaction effect. The presence of the catalyst significantly increased the FC content of the biochar at low temperature. However, the maximum FC value of 58.65% was obtained at a temperature of 723 K with without the catalyst, which justifies the significance of interaction effect. Additionally, devolatilization reduced the volatile matter in the biochar (26–70.08%) relative to the raw CCF (85.42%).

The biochar was primarily composed of carbon (48.27–67.58%), followed by oxygen (13.96–36.09%), nitrogen (10.29–19.39%), and hydrogen (2.98–5.34%). Noncatalytic pyrolysis at higher temperatures (723 K) yielded a biochar with a high carbon content, similar to that reported by Silva et al.^10^ for biochar from malt waste. According to Table 7, both temperature and MgCl_2_ concentration exhibited significant main effects on the elemental composition of biochar, generally favoring higher carbon content and lower oxygen and hydrogen contents. However, the significant interaction terms, which displayed opposite signs and comparable magnitudes, indicate that these trends are dependent on the combined levels of temperature and catalyst loading, and therefore do not apply uniformly across all experimental conditions.

Elevated pyrolysis temperatures promote the volatilization of thermally unstable compounds and the breakdown of aliphatic structures, favoring the formation of stable aromatic carbon frameworks and resulting in lower oxygen and hydrogen contents (%O and %H). The addition of MgCl_2_ enhances these effects by catalyzing dehydration reactions and suppressing the release of oxygenated species, thereby increasing carbon retention in the biochar matrix.^41^ Consequently, the combined effect of high temperature and MgCl_2_ leads to biochar with higher fixed carbon content and a more condensed aromatic structure.

These structural transformations are further illustrated in the Van Krevelen diagram presented in Figure 8, which correlates the aromaticity (H/C atomic ratio) and polarity (O/C atomic ratio) of the biochars and the raw biomass. According to Liu et al.,^11^ dehydration reactions release hydrogen and oxygen in a 2:1 molar ratio. The linear correlation between H/C and O/C (R^2^ = 0.9999; slope = 1.936) confirms that dehydration was the dominant devolatilization mechanism. This is consistent with the observed enhancement of dehydration at elevated temperatures and in the presence of MgCl_2_. As a result, the biochars exhibited lower aromaticity and polarity compared to the raw biomass, in agreement with previous studies.^10,42^

**Figure 8:**
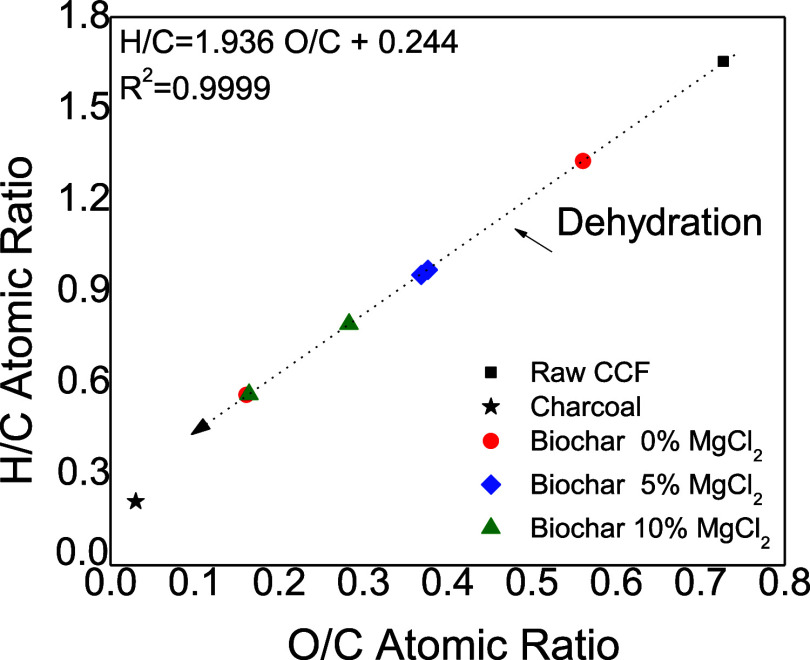
Van Krevelen diagram: H/C ratio as a function of O/C ratio, for raw CCF, charcoal, and biochar produced by the CCF pyrolysis at 0%, 5% and 10% of MgCl_2_.

The higher heating value (HHV) of the biochar ranged from 18.17 to 25.09 MJ/kg, superior to raw CCF (14.57 MJ/kg). According to Table 7, both temperature and MgCl_2_ concentration directly impact the HHV of biochar,^10^ but due to the interaction effect between the factors, the biochar with the highest HHV was produced at 723 K without the catalyst and it has a great potential for energy applications comparable to mineral coal (26.5 MJ/kg).

Figure 9 shows the FTIR spectra of biochars produced under different pyrolysis conditions. A weak band between 3000 and 3600 cm^–1^, associated with O–H stretching in water, alcohols, and phenols, decreases with increasing temperature due to the dehydration of thermally unstable hydroxyl groups.^43^ This reduction was less pronounced in MgCl_2_-treated samples, likely due to water retention by the hydrated salt.

**Figure 9:**
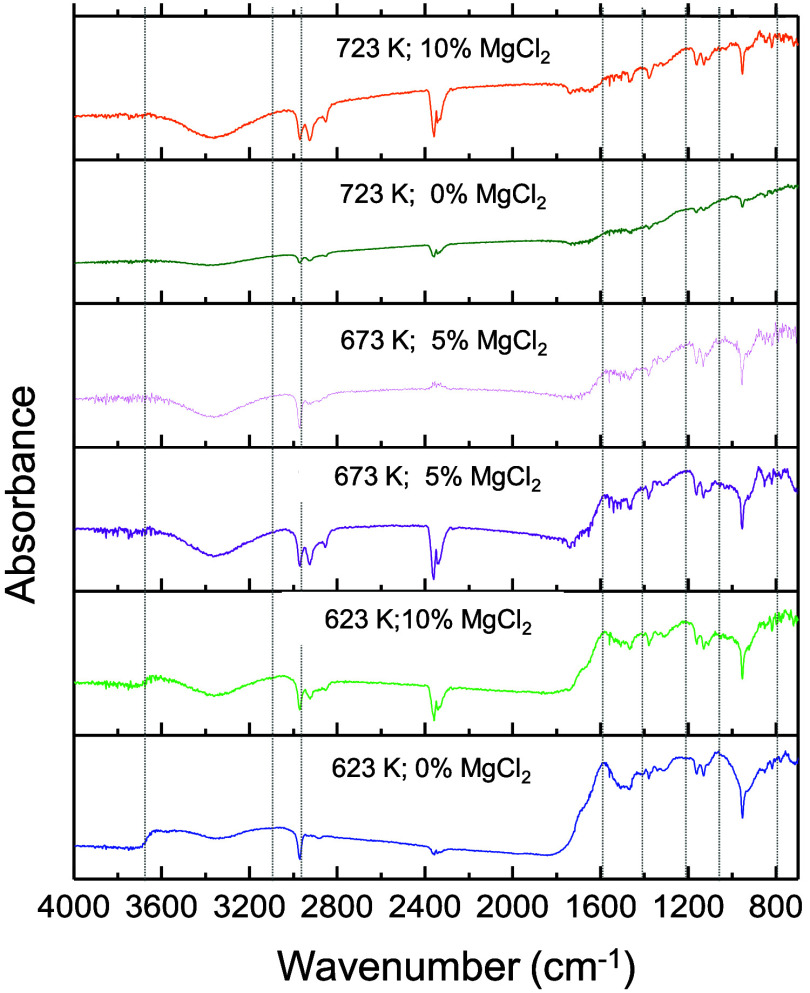
FTIR spectra of raw CCF and biochar.

The peak near 2970 cm^–1^, related to aliphatic C–H bonds from alkanes and alkenes, also diminished with temperature, reflecting the breakdown of weak C–H bonds.^44^ Aromatic C=C stretching at around 1600 cm^–1^ decreased as well, indicating progressive devolatilization at higher temperatures.^45^ In the 1410 cm^–1^ region, attributed to CH_2_ bending adjacent to carbonyl groups,^43,46^ peak intensity declined with temperature but increased with MgCl_2_, suggesting that the salt may promote carbonyl retention. Peaks near 1200 and 1020 cm^–1^, associated with C–O and C–O–C vibrations in polysaccharides and ethers,^43^ became less intense at higher temperatures, particularly at 723 K without MgCl_2_, indicating the loss of oxygenated functionalities. Finally, in the 970–700 cm^–1^ region, corresponding to aromatic C–H vibrations, signal attenuation was most evident at 723 K without the catalyst. This behavior, attributed to hydrogen loss from aromatic rings,^47^ suggests increased carbon condensation and a more ordered structure in high-temperature biochars.

Figure 10 compares the scanning electron microscopy (SEM) images of raw CCF and the biochar produced at 723 K with 0% (Run 3) and 10% MgCl_2_ (Run 4). Figure 10a shows the SEM micrograph of the raw biomass, revealing fibrous structures with a heterogeneous, rough-textured surface and limited porosity. The biochar from Run 3 (723 K; 0% MgCl_2_) presented signs of thermal transformations indicative of carbonization (Figure 10b), with the onset of pore formation becoming apparent. An increase in surface roughness and the presence of collapsed structures can be attributed to the volatilization of organic compounds during the pyrolysis process. According to Figure 10c, the biochar obtained from catalytic pyrolysis (Run 4: 723 K and 10% of MgCl_2_) exhibited enhanced porosity, with irregularly distributed crater-shaped structures resembling a beehive and microcrystals on its surface, indicating the presence of inorganic compounds, primarily magnesium-based.^10^

**Figure 10:**
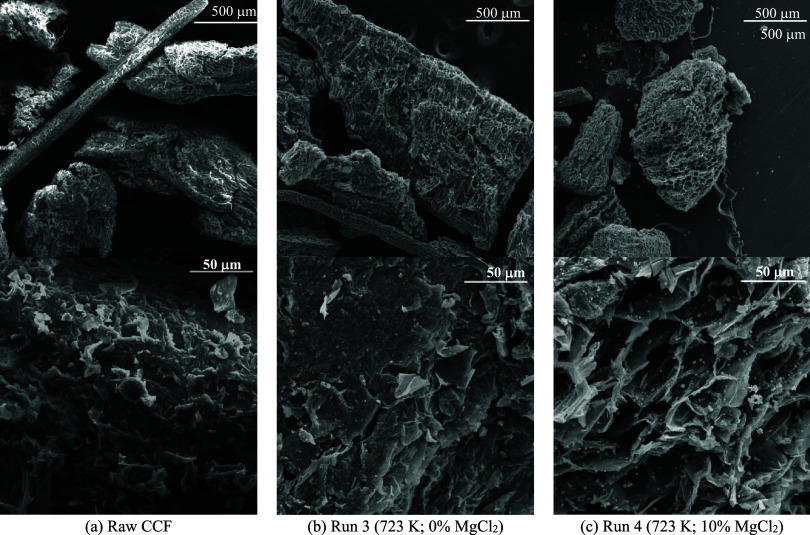
SEM images of raw biomass (a) and CCF biochar produced at 723 K with 0% of MgCl_2_ (b) and 10% of MgCl_2_ (c).

Table 8 shows the atomic concentrations of C, O, Mg, Cl, and other elements on the sample’s surfaces, obtained from SEM-EDS analysis, with ± 10% accuracy. As expected, there was an increase in carbon concentration in the biochar compared with raw biomass, associated with a strong decrease in oxygen content, due to the devolatilization. The biochar from Run 4 showed a greater content of Mg and Cl, due to the salt impregnation. It is important to note that the inorganic elements constitute the ash content, predominantly located within the pores, not on the surface. Consequently, the values obtained from the EDS analysis are qualitative.

**Table 8:** Percentage Elemental Composition Obtained by EDS from Samples of Raw Biomass, Biochars Obtained From Run 3 (450°C; 0% MgCl_2_) and Run 4 (450°C; 10% MgCl_2_)

	composition (%)					
element	raw biomass		biochar Run 3		biochar Run 4	
C	60.782	±6.214	76.223	±5.387	77.534	±2.942
O	34.957	±5.932	7.230	±2.390	7.951	±0.226
Na	0.153	±0.024	0.260	±0.054	0.287	±0.053
Mg	0.125	±0.039	0.168	±0.109	2.965	±0.184
Al	0.009	±0.022	0.029	±0.045	0.145	±0.151
Si	0.150	±0.211	0.532	±0.836	0.028	±0.048
P	0.122	±0.077	0.306	±0.084	0.208	±0.031
S	0.131	±0.098	0.382	±0.160	0.028	±0.048
Cl	1.090	±0.170	3.739	±1.556	5.317	±1.290
K	2.187	±0.724	10.375	±5.923	4.926	±1.261
Ca	0.285	±0.202	0.376	±0.233	0.415	±0.027

Regarding to the potential applications of the CCF-derived biochars, their physicochemical characteristics clearly support their suitability as soil conditioners. The fixed carbon content increased substantially with conversion severity, reaching 62.0% at 723 K, while the atomic ratios O/C (0.24–0.48) and H/C (0.87–1.25) decreased across all treatments (Figure 7), indicating higher aromaticity and structural stability, which contribute to long-term persistence in soils and enhance the potential for carbon sequestration. FTIR spectra further confirmed the retention of surface O–H and C–O groups, which are associated with cation-exchange interactions and water retention capacity, supporting their agronomic relevance.

Complementing the chemical attributes, SEM images (Figure 8) revealed a porous microstructure capable of improving aeration and moisture regulation, while the presence of residual Mg in catalyst-assisted runs suggests additional benefits such as nutrient supply and potential pH buffering. Together, the combination of elevated aromatic carbon content, moderate functional group density, and a structurally favorable morphology provides robust evidence that the biochars produced here possess key features aligned with effective soil conditioning and soil-quality enhancement, even in the absence of agronomic trials.

The biochar samples were prepared under controlled and reproducible conditions and exhibited good macroscopic homogeneity, ensured by uniform impregnation procedures, well-defined pyrolysis conditions, and thorough mixing prior to analysis. It is important to emphasize that the homogeneity referred to in this study is strictly macroscopic in nature and does not imply absolute chemical uniformity at the microscopic scale. This bulk-level homogeneity is further supported by the consistency of the proximate analysis results and the reproducibility of ash contents among the samples.

Nevertheless, the elemental compositions obtained by SEM–EDS should be interpreted as qualitative or, at most, semiquantitative information. This limitation is inherent to the EDS technique, which probes localized regions of the sample surface and whose response depends on factors such as interaction volume, surface topography, detector efficiency, and instrumental calibration. Consequently, SEM–EDS data do not necessarily represent the average elemental composition of the entire surface or the bulk material, even when the sample is macroscopically homogeneous.

At the microscale, inorganic species in biochars may still exhibit localized enrichment or depletion zones, particularly within pores or at mineral–carbon interfaces. Accordingly, SEM–EDS is employed in this work primarily to qualitatively confirm the presence and relative surface occurrence of inorganic elements, such as Mg and Cl derived from the catalyst, and to support the morphological observations obtained by SEM, rather than to provide absolute quantitative elemental concentrations. The qualitative trends observed by EDS are consistent with the ash contents derived from proximate analysis, reinforcing the reliability and internal coherence of the overall interpretation.

## Conclusions

This study systematically investigated the catalytic pyrolysis of coconut coir fiber (CCF) in a fixed-bed reactor, with emphasis on the combined effects of temperature and magnesium chloride (MgCl_2_) loading on thermal degradation behavior, reaction kinetics, product yields, and product quality.

The results demonstrated that CCF is a suitable feedstock for thermochemical conversion, yielding substantial fractions of bio-oil, biochar, and noncondensable gases under all conditions studied. In the absence of catalyst, increasing the pyrolysis temperature from 623 to 723 K favored liquid production, with bio-oil yields increasing from approximately 49.9% to a maximum of 52.36%, while biochar yields decreased from 32.49% to 29.73% and gas yields remained relatively stable at around 17–18%.

The addition of MgCl_2_ significantly altered the product distribution. At 10% MgCl_2_ loading, biochar yields increased markedly, reaching values between 37.17% at 623 K and up to 32.56% at 723 K, accompanied by a corresponding reduction in bio-oil yield. Gas yields ranged from 15.39% to 17.29%, indicating that the catalyst preferentially promoted solid-phase formation rather than gasification. These trends confirm that MgCl_2_ shifts the pyrolysis pathways toward dehydration and carbonization reactions, enhancing char formation at the expense of liquid products.

Thermogravimetric and kinetic analyses corroborated these observations, showing that MgCl_2_ substantially reduced the activation energy of CCF decomposition, from 56.3 kJ·mol^–1^ for raw biomass to 29.3 kJ·mol^–1^ at 10% MgCl_2_, thereby facilitating devolatilization at lower temperatures. Bio-oil composition analysis further revealed that MgCl_2_ favored the formation of aldehydes, particularly furfural, through enhanced hemicellulose dehydration, while higher temperatures promoted phenolic compounds derived from lignin degradation.

The produced biochars exhibited high fixed carbon contents (20.6–58.7%) and elevated heating values (18.2–25.1 MJ·kg^–1^), indicating their suitability for energy recovery and carbon sequestration applications. Overall, the results demonstrate that pyrolysis temperature primarily governs bio-oil yield, whereas MgCl_2_ loading is a key parameter for enhancing biochar production and tailoring product selectivity. These findings highlight the potential of catalytic pyrolysis as an effective strategy for valorizing coconut fiber into energy carriers and functional carbon materials.
